# Association Study of Apolipoprotein E Gene Polymorphism With Incidence and Delayed Resolution of Hemifacial Spasm

**DOI:** 10.3389/fneur.2021.760126

**Published:** 2021-12-15

**Authors:** Jianxin Zhou, Li Jiang, Sangui Yuan, Jiashang Huang, Quanhong Shi, Yanfeng Xie, Bo Deng, Yan Zhan

**Affiliations:** Department of Neurosurgery, The First Affiliated Hospital of Chongqing Medical University, Chongqing, China

**Keywords:** APOE, hemifacial spasm, polymorphism, delayed resolution, incidence rate

## Abstract

**Objective:** This study investigates the correlation between Apolipoprotein E gene (APOE) polymorphism and the incidence and delayed resolution of hemifacial spasms.

**Methods:** The APOE genotypes of 151 patients with hemifacial spasm and 73 control cases were determined by cleaved amplification polymorphism sequence-tagged sites. The distribution of three APOE alleles (ε2, ε3, and ε4) in two groups and the delayed resolution rate in 6 genotypes were calculated and statistically analyzed.

**Results:** The proportion of patients with APOE ε3/ε4 genotype in the hemifacial spasm group (25.17%) was significantly higher than that in the control group (12.33%) (*P* = 0.027). In terms of allele frequency, the proportion of the APOE ε4 allele in the hemifacial spasm group (15.56%) was significantly higher than that in the control group (6.85%) (*P* = 0.009). Meanwhile, the proportion of APOE ε4 allele carriers in the hemifacial spasm group (29.80%) was significantly higher than that in the control group (13.7%) (*P* = 0.009). Logistic regression analysis showed that the ε4 allele significantly increased the incidence of hemifacial spasm (*OR* 2.675, 95%*CI* 1.260-5.678, *P* = 0.010). Among the 32 patients with a delayed resolution, the ε3/ε3 and ε3/ε4 had the highest proportion in 6 genotypes. The delayed resolution rate of APOE ε3/ε4 (34.21%) was significantly higher than APOE ε3/ε3 (17.78%) (*P* < 0.05). The delayed resolution rate of APOE ε4 carriers was the highest (33.33%) in the 3 allele carriers, but there was no significant difference among the 3 allele carriers (*P* = 0.065).

**Conclusion:** The polymorphism of APOE is relevant to the incidence rate of hemifacial spasms. APOE ε4 allele increases the incidence of hemifacial spasm. The APOE ε4 allele may promote the occurrence of delayed resolution.

## Introduction

Hemifacial spasm (HFS) refers to a common cranial nerve disease characterized by involuntary contractions of muscles, which originates from the orbicularis oculi muscle and gradually progresses to the whole facial muscle (1). At present, the etiology is still unclear. It is generally accepted that HFS results from the vascular compression of the facial nerve at the root entry zone, and demyelination and axonal loss are the primary pathologies (2–4). However, many people with vascular compression do not show any symptoms (5). According to the pathogenesis, the symptoms of all patients should be relieved immediately after an operation, but there are still some patients present with delayed resolution. The current theories do not fully explain the above-mentioned phenomenon. Therefore, we speculate that there might be some potential risk factors that influence myelin repair.

Apolipoprotein E is a major apolipoprotein that is mapped by the apolipoprotein E gene (APOE), which has 3 alleles (APOE ε2, APOE ε3, and APOE ε4) and 6 genotypes (6). Some studies found that APOE ε4 could accelerate the pathophysiological process by interfering with remyelination (7, 8). Based on the fact that demyelination is a characteristic pathological change of HFS and plays an important role in the occurrence of disease, we speculate that APOE ε4 may promote the occurrence of HFS and delayed resolution by affecting the repair of the myelin sheath.

In this study, we aimed to explore the association of APOE polymorphism with the incidence and delayed resolution of HFS by analyzing the differences in the distribution of APOE in different populations.

## Data and Method

This retrospective case-control study included 151 patients with HFS [90 females and 61 males with an average age of 46.99 ± 7.79 (SD) years] who were admitted to the Department of Neurosurgery, the First Affiliated Hospital of Chongqing Medical University from September 2019 to March 2021. In addition, 73 control participants [38 females and 35 males with an average age of 44.72 ± 10.02 (SD) years] were included in this study as the control group. The study was approved by the Bioethics Committee of the First Affiliated Hospital of Chongqing Medical University (No. 2019-258). Informed consent was obtained from all the individual participants included in the study.

### Inclusion and Exclusion Criteria

#### HFS Group

Inclusion criteria (9): consistent with the clinical manifestation of HFS, with a history longer than six months, excluded from secondary lesions by CT or MRI. Exclusion criteria: anamnesis of facial paralysis, mental illness, cerebrovascular diseases such as cerebral infarction, encephalitis, or dementia.

#### Control Group

Inclusion criteria: the normal controls were recruited from the healthy subjects who came to our hospital for routine physical examinations. Exclusion criteria: anamnesis of cranial nerve disease, craniocerebral trauma, intracranial space occupation, cerebrovascular disease, intracranial infection, mental illness, or dementia.

### APOE Genotype Identification

A total of 2 mL of venous blood was collected from all participants, DNA was extracted by using Xinbaji nucleic acid extraction reagent. The DNA of the participants, the weak positive control group, and the blank group were added to the reaction tube that had APOE gene detection reagents (provided by Wuhan YZY Biopharma); then installed into the Applied Biosystems 7,500 Real-Time PCR system for amplification (the first stage: 37°C 10 min, 1 cycle; the second stage: 95°C 5 min, 1 cycle; the third stage: 95°C 15 s, 60°C 60 s, 40 cycles; signal acquisition: the third stage was collected FAM, VIC, and ROX signals under 60°C and enabled the Real-Time PCR). The genotype was determined according to the Ct value of the FAM channel and VIC channel, The genotyping method was shown in Table 1.

**Table 1:** Apolipoprotein E gene (APOE) genotype decision table.

**APOE genotype**	**APOE monitoring site**	
	**ε2**	**ε4**
ε2/ε2	526T/T	388T/T
ε2/ε3	526C/T	388T/T
ε2/ε4	526C/T	388T/C
ε3/ε3	526C/C	388T/T
ε3/ε4	526C/C	388T/C
ε4/ε4	526C/C	388C/C

### Grading the Degree of Spasm in Patients With HFS

The symptomatic change (SMC) grading system (Grade I: Localized spasm of the orbicularis oculi; Grade II: The spasm spreads to other ipsilateral facial muscles; Grade III: Vision is affected because of the frequent spasms; Grade IV: Disfiguring asymmetry) that developed by Kwan Park et al. (10) was used in this study to grade the degree of spasm in patients with HFS. The correlation between the degree of spasm and the course of the disease was further analyzed.

### Classification of Postoperative Symptoms

The symptoms after the microvascular decompression of HFS were divided into four types (11). Type 1: The symptoms disappeared immediately after operation; Type 2: The symptoms were relieved after an operation and gradually disappeared; within 7 days to 2 years; Type 3: The symptoms disappeared immediately after the operation, but recurred within 3 days, and then gradually disappeared and Type 4: Invalid after the operation.

### Statistical Analysis

All data were analyzed by SPSS v.22. Measured data were expressed as mean ± *SD* (*x* ± *SD*) and tested by the *t*-test. Enumeration data were tested by Pearson chi-square test or Fisher's exact test. Risk factors analysis was conducted using the logistic regression model. All tests of significance were two-sided with a *p*-value < 0.05.

## Results

### General Data

A total of 151 patients with HFS were included in this study. Among them, 78 cases were left-sided, 73 cases were right-sided. The mean course of the disease was 65.88 ± 63.99 (SD) months (range 12–360 months). As shown in Table 2, there was no statistical difference between the two groups in gender, age, and incidence of hypertension (*P* > 0.05).

**Table 2:** Comparative analysis table between the hemifacial spasm (HFS) group and the control group (*n, x* ± *s*).

**Clinical data**	**Control group** **(*n =* 73)**	**HFS group** **(*n =* 151)**	***t***/**χ^2^ value**	***P*** **value**
Gender male	35	61	1.145	0.285
Female	38	90		
Age (year)	44.71 ± 10.02	46.99 ± 7.79	1.710	0.090
Hypertension	17	40	0.266	0.606

### The Correlation Between the Degree of Spasm and the Course of the Disease

There were 29 patients with grade I HFS, 67 patients with grade II HFS,46 patients with grade III HFS, and 9 patients with grade IV HFS. Based on the SMC grading system, the mean course of the disease was 47.6 months (range: 12–180 months) in grade I, 55.3 months (range: 12–204 months) in grade II, 71.2 months (range: 7.5–240 months) in grade III, and 176 months (range: 24–360 months) in grade IV patients. We found that the longer disease duration was associated with higher SMC grade (*P* < 0.05).

### Genotype Distribution

**Figure 1:**
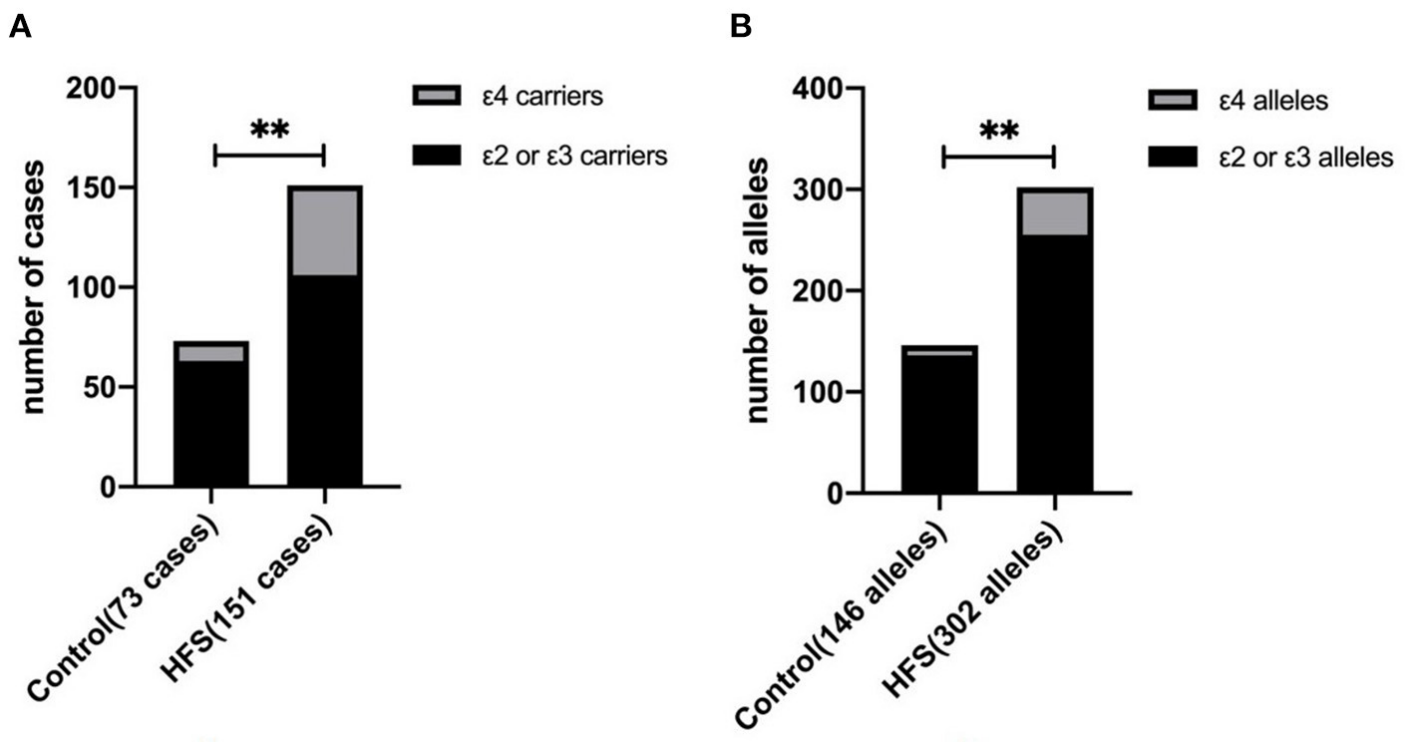
The difference between the hemifacial spasm group and the control group in the ε4 allele frequency and the ε4 allele carrier. The proportion of ε4 allele carriers in the hemifacial spasm group was higher than that in the control group **(A)**. The ε4 allele frequency in the hemifacial spasm group was higher than that in the control group **(B)**. (^**^ : *P* < 0.001).

APOE has 3 alleles (APOE ε2, APOE ε3, and APOE ε4) and 6 phenotypes (ε2/ε2, ε2/ε3, ε2/ε4, ε3/ε3, ε3/ε4, and ε4/ε4). The genotype distribution, allele frequencies, and allele carriers frequencies of patients with HFS and the control group are shown in Table 3. In the control group, there were 1 of ε2/ε2, 10 of ε2/ε3, 1 of ε2/ε4, 52 of ε3/ε3, and 9 of ε3/ε4. In the HFS group, there were 2 of ε2/ε2, 14 of ε2/ε3, 5 of ε2/ε4, 90 of ε3/ε3, 38 of ε3/ε4, and 2 of ε4/ε4. Among 6 phenotypes, the proportion of ε3/ε4 in the HFS group (25.17%) was significantly higher than that in the control group (12.33%) (*P* = 0.027). The proportion of the APOE ε4 allele carriers (ε2/ε4, ε3/ε4, ε4/ε4) in the HFS group was 29.80%, which was higher than that in the control group (13.70%) (*P* = 0.009) (Figure 1A). The frequencies of the APOE ε4 allele in the HFS group and control group were 15.56% and 6.85% (Figure 1B), the difference was also statistically significant (*P* = 0.009).

**Table 3:** Statistics Analysis of the HFS group and the control group (*n*, %).

		**Control** **group** **(*n =* 73)**	**HFS** **group** **(*n =* 151)**	**χ^2^ value**	***P*** **value**
Phenotype	ε2/ε2	1 (1.37)	2 (1.32)		1.000
	ε2/ε3	10 (13.70)	14 (9.27)	1.008	0.315
	ε2/ε4	1 (1.37)	5 (3.31)	0.162	0.688
	ε3/ε3	52 (71.23)	90 (59.60)	2.868	0.090
	ε3/ε4	9 (12.33)	38 (25.17)	4.891	0.027
	ε4/ε4	0 (0.00)	2 (1.32)		1.000
Carrier	ε2	12 (16.44)	21 (13.90)	0.251	0.616
	ε3	71 (97.26)	142 (94.04)	0.512	0.474
	ε4	10 (13.70)	45 (29.80)	6.888	0.009
Frequency of allele	ε2	13 (8.90)	23 (7.62)	0.221	0.638
	ε3	123 (84.25)	232 (76.82)	3.299	0.069
	ε4	10 (6.85)	47 (15.56)	6.729	0.009

### Single-Factor Logistics Regression Analysis–Risk Factors of HFS

A total of 45 (29.8%) of 151 patients with HFS were APOE ε4 allele carriers and 10 (13.7%) of 73 control cases were APOE ε4 allele carriers, the difference was statistically significant (*P* = 0.009). As shown in Table 4, logistic regression analysis showed that the e4 allele was found to be a significant increase in the incidence of HFS (*OR* 2.675, 95%*CI* 1.260–5.678, *P* = 0.010).

**Table 4:** Single-factor logistics regression of risk factors for HFS.

**Factor**	***b***	**SE (*b*)**	**Waldχ^2^**	***P***	***OR*** **(95% *CI*)**
ε2 carrier	−0.197	0.394	0.250	0.617	0.821 (0.380–1.777)
ε3 carrier	−0.686	0.804	0.729	0.393	0.504 (0.104–2.433)
ε4 carrier	0.984	0.384	6.560	0.010	2.675 (1.260–5.678)

### Correlation Between APOE Gene and Delayed Resolution

There were 118 cases of type 1, 24 cases of type 2, 8 cases of type 3, and 1 case of type 4 in 151 patients with HFS after the operation. One invalid patient after operation showed ε3/ε4 genotype. There were 32 patients with delayed resolution (Type 2 and 3). Among the 32 patients, the genotype frequencies were 50.00% for ε3/ε3, 40.62% for ε3/ε4, 3.12% for ε2/ε2, ε2/ε4, and ε4/ε4, 0% for ε2/ε3. Because the ε3/ε3 and ε3/ε4 had the highest proportion in 6 genotypes, we specifically compared the delayed resolution rate of these two genotypes.

**Figure 2:**
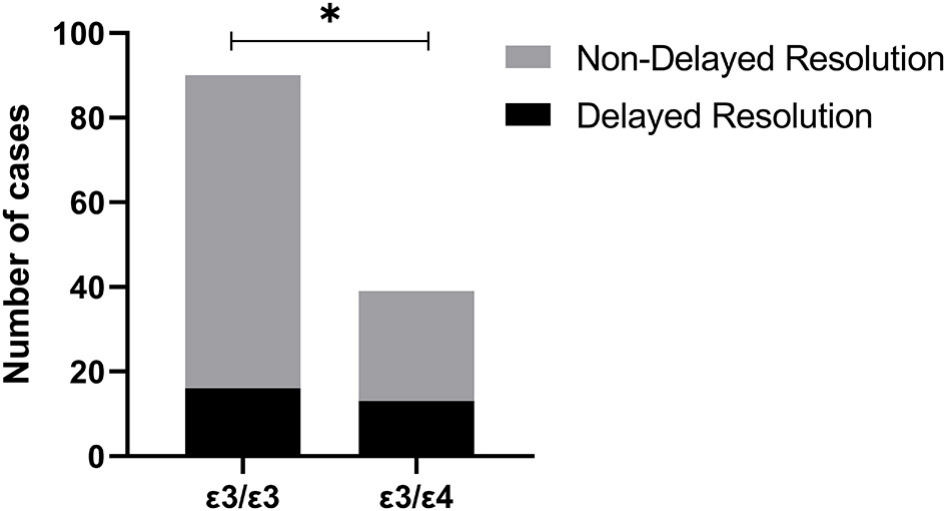
The delayed resolution rate of Apolipoprotein E gene (APOE) ε3/ε4 (34.21%) was significantly higher than APOE ε3/ε3 (17.78%) (*: *P* < 0.05).

As shown in Table 5, the differences in delayed resolution rates among the 6 genotypes were statistically significant (*P* = 0.027), the delayed resolution rate of APOE ε3/ε4 (34.21%) was significantly higher than APOE ε3/ε3 (17.78%) (*P* < 0.05) (Figure 2). The delayed resolution rate of APOE ε4 carriers was the highest (33.33%) in the three allele carriers, but there was no significant difference in the delayed resolution rate among the three allele carriers (*P* = 0.065).

**Table 5:** Analysis table of delayed resolution rate in the different APOE genotypes and allele carriers *(n, %)*.

**Phenotype**	**ε2/ε2**	**ε2/ε3**	**ε2/ε4**	**ε3/ε3**	**ε3/ε4**	**ε4/ε4**	**χ^2^ value**	***P*** **value**
Delayed resolution	1 (50.00)	0 (0.00)	1 (20.00)	16 (17.78)	13 (34.21)	1 (50.00)		0.027
Non-delayed resolution	1 (50.00)	14 (100.00)	4 (80.00)	74 (82.22)	25 (65.79)	1 (50.00)		
Total	2	14	5	90	38	2		
**Carrier**	**ε**2	**ε3**	**ε4**				**χ^2^ value**	***P*** **value**
Delayed resolution Non-delayed resolution Total	2 (9.52) 19 (90.48) 21	29 (20.42) 113 (79.58) 142	15 (33.33) 30 (66.67) 45				5.457	0.065

## Discussion

The APOE gene consists of four exons and three introns, totaling 3,597 nucleotides. APOE has 3 alleles (APOE ε2, APOE ε3, and APOE ε4) and 6 phenotypes (ε2/ε2, ε2/ε3, ε2/ε4, ε3/ε3, ε3/ε4, and ε4/ε4). Among them, APOE ε3 is the wild type that has normal function and the highest proportion in the population (12, 13). APOE ε2 and ε4 are mutated, ε4 is recognized as a negative regulator in many diseases. Studies have also shown it can increase the risk of developing early-onset and sporadic Alzheimer's disease (14–16). Other research considered that APOE polymorphism might affect the prognosis of traumatic brain injury patients, and the prognosis of ε4 carriers is worse (17, 18). Besides, ε4 might promote the release of inflammatory factors (19), and ε4 also plays a contributory role in the pathological processes of cerebrovascular diseases and demyelinating diseases (20–23).

This study shows that the proportion of patients with APOE ε3/ε4 genotype in the HFS group (25.17%) is significantly higher than that in the control group (12.33%) (*P* = 0.027). In terms of allele, the proportion of APOE ε4 allele in the HFS group (15.56%) is significantly higher than that in the control group (6.85%) (*P* = 0.009). Meanwhile, the proportion of APOE ε4 allele carriers in the HFS group (29.80%) is significantly higher than that in the control group (13.7%) (*P* = 0.009). And the logistic regression analysis shows that the ε4 allele significantly increases the incidence of HFS. Thus, we speculate that there could be a correlation between APOE polymorphism and the incidence of hemifacial spams. APOE ε4 allele carriers are more likely to develop HFS compared with APOE ε3 and APOE ε2 allele carriers. However, the specific mechanism of APOE ε4 in promoting the initiation of HFS remains unknown.

At present, the etiology and pathogenesis of HFS are still inconclusive, there are three existing theories to explain the pathogenesis. Some researchers believe that due to the lack of Schwann cells in the root exit zone of the facial nerve, long-term compression of adjacent vessels leads to a focal demyelinating lesion. The ectopic electrical activity that is transmitted across synapses occurs between the exposed axons of nerve fibers, which eventually leads to the involuntary twitch of facial muscles (24, 25). Other researchers believe that the facial nerve produces an antidromic conduction electrical signals due to the vascular compression in the root exit zone, it increases the excitability of the facial nerve nucleus and changes its function and structure, forming an explosive downward transmission electrical activity and causing the clinical symptoms (26, 27). The sympathetic theory (28–30) proposed by Chinese researchers believes that the long-term vascular compression in the root exit zone leads to the damage of the vessel wall and the demyelination of the facial nerve. The sympathetic nerve fibers that are exposed in the vessel wall are in direct contact with the exposed facial nerve, which has become a bridge between the different facial nerve fibers. When the sympathetic nerves are excited, the released neurotransmitters will act on the damaged facial nerve and make it generate action potentials. The electrical signals then spread to other branches of the facial nerve through the sympathetic nerve bridge and resulting in clinical symptoms. Therefore, HFS may be related to the demyelinating lesion, the hyperexcitability of the facial nucleus, the release of neurotransmitters, and so on.

However, many imaging and autopsy findings of posterior cranial fossa confirm that neurovascular compression also exists in the normal population (5), while not all cases develop HFS. Therefore, we suppose that there are some underlying mechanisms to promote HFS. Based on prior studies, APOE ε4 was considered to affect the pathophysiological processes of several diseases by participating in multiple important signaling pathways. Some studies found that APOE ε4 could accelerate pathophysiological processes by interfering with neurons and remyelination (7, 8). Pathological changes such as demyelination, axon loss, and myelin hyperplasia could be found in patients with HFS (4). We then speculate that the repair of myelin sheath in the compressed area of nerves interfered in the ε4 gene carriers, thus the heterotopic conduction of electrical signal is more likely to occur. APOE ε4 is possibly related to neuro-excitatory toxicity caused by the excessive release of excitatory amino acids (31). It was also found that APOE ε4 has no anti-inflammatory as APOE ε2 and APOE ε3 do. Instead, APOE ε4 promotes the secretion of pro-inflammatory and exacerbates neuroinflammation (32, 33). Interleukin-6 and other inflammatory cytokines in HFS patients are found to be a higher percentage than that in normal people (34). Therefore, APOE ε4 might cause HFS by promoting the release of inflammatory factors, with which patients are more prone to HFS than that with APOE ε2 and APOE ε3. Still, we need further theoretical basis and clinical research to corroborate the above hypothesis.

At present, the exact pathogenesis of delayed resolution after microvascular decompression is still unclear. Moller and Hatem (35, 36) proposed that in patients with a delayed resolution, it took some time for the recovery of demyelination changes and the high excitability of the facial nerve nucleus. Even if vascular compression has been relieved, the myelin regeneration and recovery of facial nucleus excitability still need a period. Kwan et al. (37) reported that the third month after surgery was the earliest time to predict the surgical outcome for delayed resolution patients. Due to the APOE ε4 gene could affect the repair of the myelin sheath, according to the mechanism of the delayed resolution, we speculated that the incidence of demyelination changes and postoperative delayed resolution in APOE ε4 carriers' population should be higher than that in APOE ε2 or ε3 carriers. In this study, we observed that the delayed resolution rate of APOE ε3/ε4 (34.21%) was significantly higher than that of APOE ε3/ε3 (17.78%) (*P* < 0.05). This finding strongly corroborates our hypothesis. The delayed resolution rate of APOE ε4 carriers was the highest (33.33%) in the three allele carriers, but the difference was not statistically significant. As a single-center study, the sample size of this research is small. A larger sample group and multi-center study are needed for further verification of the role of APOE ε4 in the delayed resolution of HFS.

This study shows that the APOE ε4 allele increases the incidence of HFS. We speculate that due to the APOE ε4 allele promoting secretion of pro-inflammatory and exacerbating neuroinflammation, the repair of the myelin sheath at the compression area was interfered with, thus the ectopic conduction of electrical signals comes easier. And we speculate that the APOE ε4 allele may promote the occurrence of the delayed resolution, but larger sample size studies are needed for further verification. This study puts forward new evidence to further clarify the pathogenesis and prognosis of HFS.

## Data Availability Statement

The original contributions presented in the study are included in the article/Supplementary Material, further inquiries can be directed to the corresponding author/s.

## Ethics Statement

The studies involving human participants were reviewed and approved by Bioethics Committee of the First Affiliated Hospital of Chongqing Medical University (No. 2019-258). The patients/participants provided their written informed consent to participate in this study.

## Author Contributions

JZ and YZ contributed to conception and designed research. SY, LJ, QS, and BD organized the database and performed the statistical analysis. JZ wrote the first draft of the manuscript. YZ responsible for final revision. YX, QS, JH, and LJ wrote sections of the manuscript. All authors contributed to manuscript revision, read, and approved the submitted version.

## Conflict of Interest

The authors declare that the research was conducted in the absence of any commercial or financial relationships that could be construed as a potential conflict of interest.

## Publisher's Note

All claims expressed in this article are solely those of the authors and do not necessarily represent those of their affiliated organizations, or those of the publisher, the editors and the reviewers. Any product that may be evaluated in this article, or claim that may be made by its manufacturer, is not guaranteed or endorsed by the publisher.
